# Evaluation of the Efficacy and Synergistic Effect of α- and δ-Tocopherol as Natural Antioxidants in the Stabilization of Sunflower Oil and Olive Pomace Oil during Storage Conditions

**DOI:** 10.3390/ijms24021113

**Published:** 2023-01-06

**Authors:** Vassilis Athanasiadis, Theodoros Chatzimitakos, Dimitrios Kalompatsios, Dimitrios Palaiogiannis, Ioannis Makrygiannis, Eleni Bozinou, Stavros I. Lalas

**Affiliations:** Department of Food Science and Nutrition, University of Thessaly, Terma N. Temponera Street, 43100 Karditsa, Greece

**Keywords:** tocopherols, vitamin E, synergistic effect, molar ratio, antioxidant activity, olive pomace oil, sunflower oil, oxidation, lipids

## Abstract

Tocopherols are natural bioactive compounds with several health benefits. This study evaluated the effect of different ratios of α- and δ- tocopherol homologs to protect sunflower oil (SO) and olive pomace oil (OPO) against oxidation. A synergistic effect was recorded when the two tocopherols were combined at a ratio of 7:1 (α-T/δ-T). The oil samples were exposed to accelerated oxidation conditions using a Rancimat (90 °C and airflow of 15 L/h for 24 h) and protection from tocopherols was compared with that from butylated hydroxytoluene (BHT). Assessment of oil stability was examined using well-known parameters such as peroxide value (PV), thiobarbituric acid reactive substances (TBARS), *p*-anisidine value (*p*-AV), conjugated dienes (CD) and trienes (CT), and total oxidation (Totox) value, which were all significantly reduced when tocopherols were added at a ratio of 7:1 α-T/δ-T. Primary oxidative compounds measured according to PV were only reduced in SO samples (6.11%). Off-flavor compounds measured via TBARS assay in SO samples were reduced by above 20%, while *p*-AV was also reduced. CD_value_ was correlated with PV in SO samples, while the 7:1 mixture was more effective than BHT for CT_value_. Total oxidation values in SO samples and OPO samples were reduced by 6.02% and 12.62%, respectively. These values in SO samples also provided a remarkable correlation (R^2^ > 0.95) with incubation time. Moreover, the synergistic effect was not only effective in reducing the oxidation values of oil samples, but also in lowering the degradation rate of tocopherols. Protective effects from tocopherols were mainly observed in SO samples, as OPO samples were more resistant to oxidation processes. This effect was even observed in fatty acid analysis, where the 7:1 mixture provided better results than BHT-spiked samples. Thus, it is suggested that tocopherol mixtures might be used as a natural preservative in the food industry to restrain lipid oxidation processes.

## 1. Introduction

Edible oils are essential in the human diet because they not only have a high calorific value, but also provide a wide range of nutrients, such as unsaturated fatty acids and vitamins [1]. Consumption of unsaturated fatty acids is associated with maintenance of low cholesterol levels and protection against cardiovascular diseases [2]. However, the presence of double bonds in their composition renders them less stable than saturated fatty acids, making them more vulnerable to rancidity. Exposure of oils to light, heat and oxygen promotes the oxidation of fatty acids and, as a result, the nutritional value of oils deteriorates [1]. Given that molecular oxygen is a molecule that can transfer electrons from atom to atom, it can create oxygen-containing free radicals known as reactive oxygen species (ROS). ROS are highly reactive and, therefore, can rapidly react with nearby molecules [3]. Fatty acid oxidation by ROS is a complicated process, after which several toxic compounds are formed. Primary oxidation products (mostly peroxides) are unstable and can quickly degrade to secondary oxidation products, such as aldehydes and ketones, resulting in an off-flavor and displeasing taste in edible oils [4]. The production of such compounds not only may be hazardous for human health, but also has a negative impact on the quality of oils [5].

Antioxidant compounds are essential for halting the oxidation of edible oils caused by ROS. They can scavenge ROS and prolong the shelf lives of food products [6]. To minimize the detrimental effects of ROS in the food industry, antioxidants are used as food preservatives. To this end, not only can synthetic antioxidants, such as butylated hydroxyanisole (BHA) or butylated hydroxytoluene (BHT), be used, but also natural antioxidants obtained from plants. Regarding the use of synthetic antioxidants in food products, several health concerns were raised [7]. As a result, synthetic antioxidants were prohibited in numerous countries, and their use is limited. Therefore, more and more research is carried out on natural antioxidants. In addition, natural antioxidants are gaining ground over synthetic ones because consumers perceive them as healthier alternatives [8].

Vitamin E is a natural antioxidant compound consisting of four homologs (α-, β-, γ- and δ-tocopherol). It is an integral part of the human diet that exhibits multiple health benefits [9]. Tocopherols can be used as food additives due to their efficient oxidative reaction chain-breaking ability, rendering them suitable for protection against lipid oxidation or autoxidation processes [10]. The most commonly used homolog in the food industry is α-tocopherol, which exhibits the highest antioxidant activity. However, α-tocopherol can sometimes behave as a prooxidant when inserted directly into edible oils [11]. Many studies suggest that the antioxidant capacity of tocopherols is boosted when mixed with other compounds, via either synergistic or additive effects [12,13,14]. Marinova et al. investigated the synergistic effect of myricetin and α-tocopherol and the protection their mixture offers towards sunflower oil samples heated to 100 °C. They found that α-tocopherol boosts the potent antioxidant myricetin, hindering the autoxidation of triacylglycerols [13]. Lama-Muñoz et al. combined 3,4-dihydroxyphenylglycol with the natural antioxidants hydroxytyrosol and α-tocopherol in different concentrations, and spiked sunflower oil samples. These mixtures were found to be more effective at slowing the oxidation process compared to the individual compounds [14]. Therefore, studying such combinations may result in more potent antioxidant systems.

Although many combinations of tocopherols with other compounds are exploited, to the best of our knowledge, no studies have been published regarding the use of tocopherol combinations. In this study, the synergistic antioxidant activity of an α- (α-T) and δ-tocopherol (δ-T) system was examined. As per the studies by Bourgeois et al. and Von Pongracz et al., the antioxidant capacity of tocopherols is ranked δ > γ>α > β at temperatures ranging from 80 to 120 °C and α > γ>β > δ at temperatures between 20 and 60 °C. During an induction period procedure (Rancimat method) with temperature set at 90 °C, δ-T acted as a highly active antioxidant by increasing the induction period [15,16]. As a consequence, α-T and δ-T were the two homologs chosen to be investigated for their synergistic effect. A molar ratio of the two compounds was first examined. After the optimum combination was found, the mixture was evaluated in terms of its protection against the oxidation of two edible oils, sunflower oil (SO) and olive pomace oil (OPO). Since α-T is the main tocopherol contained in oils, the samples were spiked only with an appropriate amount of δ-T, so as to achieve the optimum ratio between the two. The oils were examined in terms of oxidative stability and results were compared with those of oils spiked with BHT.

## 2. Results and Discussion

### 2.1. DPPH Assay and Tocopherol Mixture Optimization

Lipid peroxidation involves a complicated series of processes, and hardly any individual antioxidant is efficient for all phases. It may be preferable to utilize a combination of antioxidants in which the compounds work synergistically [17]. The first step was to determine the optimum molar ratio between the two tocopherols (α-T and δ-T), so as to achieve maximum antioxidant activity. To gain a better overview, a 3 × 3 central composite design methodology was used. The models were verified by conducting experiments under the predicted optimal conditions and comparing the predicted values from each model with the actual (measured) values. Measured and predicted response values were recorded for each design point (Table 1). The antiradical capacity indicates that the best molecular ratio of tocopherols is 7:1 α-T/δ-T (497.8 μM TEAC) and the second best is 4:1 α-T/δ-T (422.4 μM TEAC).

The second-order polynomial equation (model), statistical parameters, and coefficients produced for the model are displayed in Equation (1) below:

*Y* = 338.02 + 55.01*X*_1_ − 41.48*X*_2_ − 3.6*X*_1_^2^ + 3.1*X*_2_^2^ − 0.73*X*_1_*X*_2_ (R^2^ = 0.99, *p* = 0.0006) (1)

**Figure 1:**
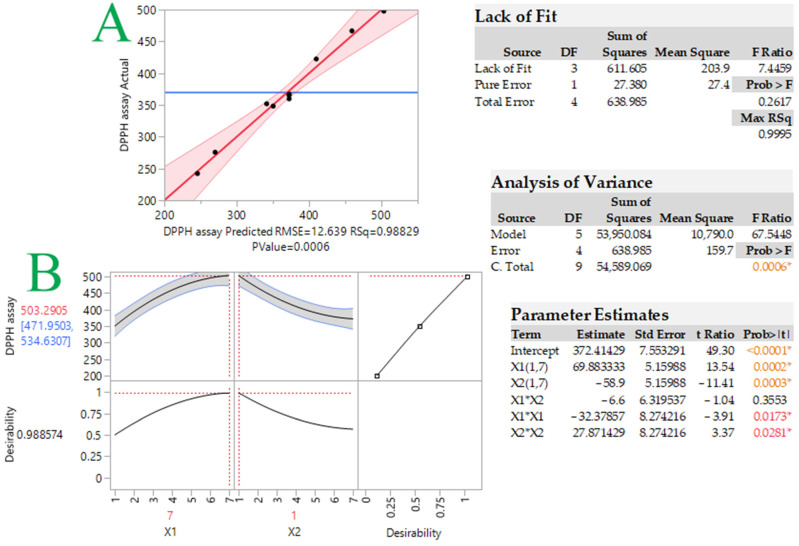
Plot comparing actual vs. predicted values for response (DPPH assay, μM TEAC) (plot **A**) and desirability function (plot **B**) for DPPH-optimized radical scavenging activity of tocopherol mixtures. Statistics for evaluation of the model that was developed are provided in the inset tables. Asterisks and colored values indicate statistically significant values.

**Figure 2:**
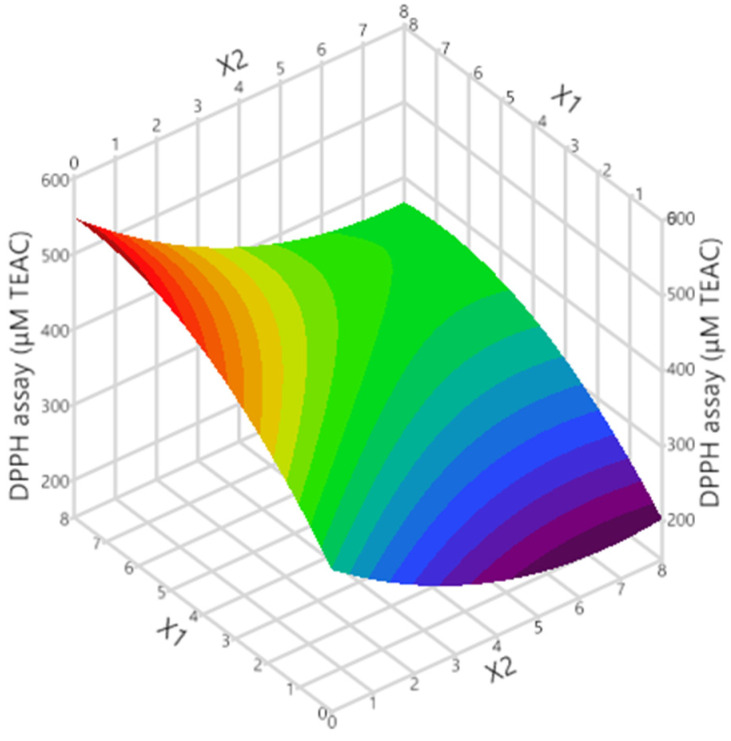
3D graph showing the impact of the considered process variables on the response.

Figure 1 and Figure 2, respectively, show the graphs of actual versus predicted response, desirability function, and 3D response. As can be observed, the coefficients were >0.99, indicating that the created models fit the data well. The optimal tocopherol combination molar ratio was found using the desirability function, and synergistic antioxidant activity should occur in the 7:1 α-T/δ-T mixture. The predicted DPPH assay was calculated to be 503.3 ± 31.3 μM TEAC under these conditions. In order to examine whether the observed effect was due to synergistic or additive effects, the antioxidant activity of the individual tocopherols at the given concentrations was examined along with that of the tocopherol mixture. Moreover, the theoretical sum of the two individual responses was calculated. Results are given in Table 2. As can be seen, the measured TEAC of the tocopherol mixture (497.8 μM TEAC) was found to be higher (statistically significant for *p* < 0.05) than the theoretical sum of the two individual responses (464.8 μM TEAC). This is indicative of synergism between these two tocopherol homologs, not an additive effect. The synergistic effect is probably based on the repair and regeneration mechanism. Potent antioxidants could be reformed using weaker antioxidants [18]. Wang et al. [19] investigated the synergistic effect of α-T with alkyl gallates, which have a similar chemical structure to tocopherols. It was found that α-T was reformed.

### 2.2. Oil Tocopherol Contents

The α-T and δ-Τ tocopherol contents in both SO and OPO samples over 24 h of Rancimat incubation time are shown in Table 3. It can be concluded that α-T was more susceptible to oxidation and was degraded much faster than δ-Τ. In SO samples, the control sample contained ~56% less α-T after 24 h of Rancimat incubation while δ-T was decreased by ~25% (statistically significant for *p* < 0.05). When the molar ratio of the two tocopherols in the oil was adjusted to 7:1 (α-T/δ-T), the contents of α-T and δ-T were decreased by ~46% and ~33% respectively (statistically significant for *p* < 0.05). In OPO samples, the contents of α-T and δ-T relative to control samples were decreased by ~40% and ~29%, respectively. In the 7:1 α-T/δ-T mixture containing OPO, α-T was decreased by ~31%, which is lower than the control sample (statistically significant for *p* < 0.05), while the δ-T content was decreased by ~66%. The synergism between the tocopherols protects them from the oxidation process. Player et al. found similar results regarding tocopherol degradation rates in soybean oil samples. These authors studied the degradation of α-, γ-, and δ- tocopherols in soybean oil at 50 °C during 24 days of storage. They concluded that α-tocopherol was degraded more easily than the other two homologs during storage time [20]. Similar results were found by Chloe [21]. Light and temperature effects on tocopherol content during oxidation of sunflower oil were studied. The results showed that γ-tocopherol was more stable than the α- homolog. Tocopherols degraded during the accelerated oxidation process by donating hydrogen from the phenol group to the lipid peroxy radical [22]. α-T exhibits high antioxidant activity in vegetable oils; however, it exhibits lower stability during storage [22,23]. It could be suggested that peroxy radical hydrogen donation seems to trigger the decline in tocopherol content. α-T has the lowest bond dissociation enthalpy (76 kcal/mol) of all tocopherol homologs of hydroxyl on the chromanol ring [24].

### 2.3. Oil Fatty Acid Compositions

Alterations in fatty acid composition are of utmost importance in oil quality, as it is associated with the degree of oxidation [23]. The composition of fatty acids in SO and OPO samples relative to incubation time can be seen in Table 4. The sum of saturated (SFA), monounsaturated (MUFA), and polyunsaturated fatty acids (PUFA) changed over time, but not in a statistically significant (*p* > 0.05) manner for any oil samples. In addition, the PUFA:SFA ratio did not change significantly in both SO and OPO samples, but it should be noted that this value is much higher in SO samples than in OPO. The MUFA:PUFA ratio barely changed over time in both oils. After 24 h of Rancimat incubation in SO samples, a higher value was observed in spiked samples, while in OPO samples the opposite could be observed. MUFA:PUFA ratio values are significantly higher (*p* < 0.05) in OPO than in SO samples. We observed significant differences in the ω-6:ω-3 ratio after 24 h of Rancimat incubation in both SO and OPO samples. The two tocopherol mixtures provided better results than the potent antioxidant BHT. The 4:1 mixture reduced the ratio by 14.48% in SO and 67.39% in OPO. The 7:1 mixture reduced the ratio by 7.80% in SO and 61.46% in OPO. An interesting finding was that the tocopherol mixtures affected the ω-6:ω-3 ratio and had statistically significant differences (*p* < 0.05) only when the oil samples were excessively exposed. This phenomenon was more intense in the OPO samples. Tocopherols do not disrupt the natural composition of oils; they respond only after prolonged oxidation processes. An ω-6 to ω-3 fatty acid ratio ranging from 1:1 to 5:1 is regarded as the most favorable for human health [25,26]. Unfortunately, during the past few decades, a noticeable decrease in the consumption of ω-3 PUFAs has emerged along with a corresponding rise in ω-6 PUFAs. Due to this, the ratio of ω-6 to ω-3 fatty acids has increased from 1:1 to 20:1 in the Western diet, compared to 45:1 in the South Asian diet [27]. It can be concluded that, between SO and OPO, the latter is healthier to consume. Calculated oxidizability value (COX) was also measured using the method introduced by Hatemi and Hammond [28], as shown below in Equation (2). Nevertheless, no significant difference (*p* > 0.05) was found in COX values between samples.

(2) $$\mathrm{COX}=\frac{1\;\left(18:1,\;\%\right)+10.3\;\left(18:2,\;\%\right)+21.6\;(18:3,\;\%)}{100}$$

### 2.4. Lipid Oxidation Indices

#### 2.4.1. Peroxide Value (PV)

Primary oxidation effects are caused by the reaction of unsaturated fatty acids with molecular oxygen and the generation of primary oxidation products, consisting of peroxides and conjugated dienes. The peroxide value method was applied to evaluate the oxidation states of oils after the Rancimat process (mostly indicating primary oxidation) [4]. The peroxide value results are given in Table 5. Generally, the more the oils were exposed to oxygen and temperature, the higher their PV. The synthetic and more potent antioxidant BHT was most effective at protecting the oils from the oxidation process, as it absorbs the electrons created by ROS. As expected, the mixture of α-T and δ-T protected oils from oxidation better than the control samples. In the control SO sample, the PV was measured to be 82.43 mmol H_2_O_2_/Kg after 24 h of Rancimat incubation. The two tocopherol-spiked samples (7:1 and 4:1 α-T/δ-T) recorded PV reductions of 6.11% and 0.77%, respectively, while the BHT-spiked SO sample had a staggering 23.40% reduction in oxidation. The reduced PV in the 7:1 tocopherol mixture suggests that more efficient prevention of oxidation occurred. Contrarily, according to PV values, OPO samples exhibited better oxidative stability than SO samples. The high amount of monounsaturated fatty acids in OPO could elicit considerable protection of the samples from oxidation [29]. After 24 h of Rancimat incubation, the highest PV was 29.37 mmol H_2_O_2_/Kg, statistically significantly lower (*p* < 0.05) than any PV from SO samples at that time. Ben-Ali et al. studied the use of methanolic basil extract as an alternative antioxidant to stabilize sunflower oil samples during accelerated storage (70 °C for 24 d). The maximum PV from the control sample was 87 mmol H_2_O_2_/Kg oil and decreased by 47.55% when spiked with 200 ppm of this extract [30].

#### 2.4.2. Thiobarbituric Acid Reactive Substances (TBARS) Assay

Secondary oxidation products are assessed by the measurement of low-molecular-weight products, consisting of aldehydes and ketones. The TBARS assay was used to determine these compounds and the results were expressed as malondialdehyde equivalents (MDAE) [4]. TBARS assay results are shown in Table 6, based on which a linear increase in TBARS value was recorded as the Rancimat incubation time increased. All control samples had the highest TBARS values. After 24 h of Rancimat incubation, TBARS values were measured to be 2.15 and 1.05 mmoL MDAE/Kg for control SO and OPO, respectively. In SO samples, 7:1 and 4:1 tocopherol-spiked samples recorded 24.65% and 21.86% reductions in TBARS values, respectively, while the BHT-spiked SO had a 31.62% reduction. This is an encouraging finding because off-flavor compounds are reduced via the addition of a small quantity of δ-tocopherol. In OPO samples, the overall TBARS values were lower than in the SO samples. The effect of tocopherols was less pronounced in OPO samples (about 10%). However, the combination of OPO monounsaturated fatty acids with BHT recorded the best reduction in TBARS value after 24 h of Rancimat incubation (60.95%). Wang et al. investigated the effect of essential oil from *Punica granatum cv. Heyinshiliu* peels when inserted into sunflower oil samples. They exposed these samples at 65 °C for 24 h and noticed that 800 ppm of the extract could significantly decrease the TBARS value of sunflower oil from 1.58 to 0.68 mg/Kg oil [31].

#### 2.4.3. *p*-Anisidine Value (*p*-AV)

*p*-AV was used to evaluate carbonyl compounds in the oil samples. Around 50% of the volatiles formed during lipid oxidation are aldehydes [32]. The results, in Table 7, showed that *p*-AV was related to the Rancimat incubation time and that tocopherols resulted in lower *p*-AV values. In the control SO sample, the *p*-AV ranged from 7.09 to 9.42, and in the control OPO sample from 5.75 to 7.35. The 4:1 α-T/δ-T mixture was found to be slightly more effective than the other tocopherol mixture. With the 4:1 mixture, the secondary oxidation products were reduced by 6.26% and 5.17% in SO and OPO samples after 24 h of Rancimat incubation, respectively. In SO samples and after 24 h of Rancimat incubation, both tocopherol mixtures had statistically significant differences (*p* < 0.05) in terms of reducing the *p*-AV. Our results are comparable to those of Chong et al., who studied the antioxidant efficacy of mango peel extracts and inserted them into sunflower oil (SO) samples. These samples were stored under accelerated conditions at 65 °C for 24 d, spiked with 100 or 200 ppm of mango peel extracts, and the results were compared with 200 ppm of synthetic BHA and 200 ppm of α-tocopherol. The maximum *p*-AV of the control SO sample was measured at 12.24 ± 0.02 and that of the 200 ppm mango peel extract-spiked SO sample was 12.06 ± 0.03, which proved to be more efficient than the other two antioxidants [33].

#### 2.4.4. Determination of Conjugated Dienes and Trienes 

Conjugated dienes and trienes are produced during primary oxidation. Their respective values (CD and CT) are linearly correlated with PV and are proportionate to the quantity of peroxides [4]. The results are shown in Table 8. In OPO samples, CD_value_ ranged from 15.69 to 19.79 mmol/Kg and CT_value_ from 3.73 to 4.86 mmol/Kg oil; no statistically significant differences were observed (*p* < 0.05). In SO samples, no statistically significant differences (*p* < 0.05) were measured in CT_value_ (8.69–12.88 mmol/Kg oil). According to Maskan and Bagci, CD_value_ and PV may not correlate in sunflower oil samples during thermal processing due to the decomposition of peroxides at high temperatures [34]. Nonetheless, CD_value_ had a wide range (13.73–40.68 mmol/Kg oil) and was correlated with PV. In SO samples, it should be noted that the 7:1 tocopherol mixture provided a slight reduction in CT_value_ (6.17%), but it protected the oil better than synthetic BHT. In OPO samples, the 4:1 mixture provided a reduction of 14.60% in CT_value_. In the study mentioned previously, Ben-Ali et al.’s results are comparable to ours for both CD_value_ and CT_value_ [30].

#### 2.4.5. Totox Value

**Figure 3:**
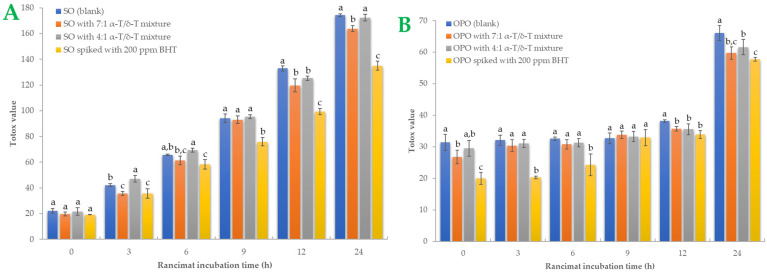
Sunflower oil (plot **A**) and olive pomace oil (plot **B**) samples’ Totox values during Rancimat incubation are shown. Standard deviation is shown with error bars, and means with different superscript letters (e.g., a–c) are statistically significantly different (*p* < 0.05) for the same Rancimat incubation time.

**Figure 4:**
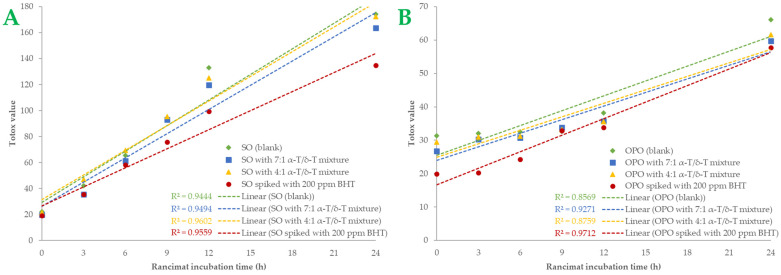
Totox values of the samples of sunflower oil (plot **A**) and olive pomace oil (plot **B**) are shown as linear curves over the Rancimat incubation time (h). Each sample’s R-squared (R^2^) is shown.

A more comprehensive index of oil oxidation is the total oxidation value, known as “Totox” value, which combines total polar compounds index with *p*-AV to provide a measurement of both primary and secondary oxidation products [5,35]. Greater oxidative stability is shown by a lower Totox value [36]. Totox value was calculated as the sum of PV value (mmoL H_2_O_2_/Κg Oil) plus *p*-AV (i.e., TV = 2 × PV + *p*-AV). The results are shown in Figure 3. Totox values in SO samples are higher than in OPO samples. By the end of the accelerated oxidation condition, SO samples had Totox values ranging from 135.03 to 174.29. The 7:1 tocopherol mixture recorded a 6.02% total reduction in oxidation products, while the 4:1 mixture reduced Totox value by 1.07%. In OPO samples, Totox values ranged from 57.75 to 66.08. The 7:1 tocopherol mixture reduced oxidation products by 12.62% and the 4:1 mixture by 6.76%. Statistically significant differences *(p* < 0.05*)* were found in BHT-spiked samples. BHT was the most effective antioxidant in both oil samples, especially in SO samples. It is known that OPO is more resistant to the oxidation process due to its MUFA content [29]. Furthermore, it could be presumed that the 7:1 tocopherol mixture is more effective than the 4:1 mixture in both SO and OPO samples. Sun-Waterhouse et al. [37] studied the encapsulation effect combined with a caffeic acid spike in olive oil samples. At 37 °C and after 30 days of storage, a vast reduction of 24.34% in Totox value (with PV expressed as meq/kg oil) was measured in encapsulated and spiked samples. In another investigation [38], variations in Totox values under accelerated conditions were remarkably comparable to those of PV. We also noticed that both oils had a linear correlation with Rancimat incubation time. The correlation coefficient (R^2^) of Totox value with Rancimat incubation time is shown in Figure 4A,B. Excellent correlation with R^2^ almost above 0.95 was measured in SO samples, and above 0.85 in OPO samples. Such a high correlation between these variables could be much more useful when it comes to predicting Totox values under specific Rancimat conditions in silico.

Based on the above, it can be speculated that molecular interactions between tocopherol isomers and ROS or lipoperoxides may occur, resulting in an overall reduced oxidation rate in oils.

## 3. Materials and Methods

### 3.1. Reagents

Tocopherol standards and malondialdehyde were purchased from Merck Ltd. (Darmstadt, Germany). Ammonium iron (II) sulfate, thiobarbituric acid, trichloroacetic acid, hydrochloric acid (37%), and glacial acetic acid were purchased from Panreac (Barcelona, Spain). Trolox (6-hydroxy-2,5,7,8-tetramethylchroman-2-carboxylic acid) was purchased from Glentham Life Sciences (Corsham, UK). Dichloromethane and isooctane were obtained from Carlo Erba (Vaul de Reuil, France). Ammonium thiocyanate and chloroform were purchased from Penta (Prague, Czech Republic). Cyclohexane and *p*-anisidine were obtained from Sigma-Aldrich (St. Louis, Burlington, MA, USA). Ethanol (99.8%) was bought from Fischer Scientific (Loughborough, UK). Hydrogen peroxide (35%) was obtained from Chemco (Malsch, Germany).

### 3.2. Materials

Oil samples were purchased from a local market in Karditsa city (Greece). The sunflower oil (SO) and olive pomace oil (OPO) were produced by the same food company. These two oil samples were selected since they have a lower market value than other oil types, such as olive oil. Therefore, they are more widely used in the mass production of food. As such, increasing their oxidative stability is of high importance.

### 3.3. Tocopherol Mixture Optimization

Pure tocopherols and tocopherol mixtures were prepared in ethyl acetate at concentrations of 200 mg/L. To investigate the interactions between α-tocopherol (α-Τ) and δ-tocopherol (δ-Τ), a 3 × 3 central composite design methodology was used. Thus, the α-T concentration (mg/L), designated *X*_1_, and the δ-Τ concentration (mg/L), designated *X*_2_, were chosen as the two independent variables. Both independent variables were coded between −1 (lower limit) and +1 (upper limit) in a central composite experimental design with two central points. These mixtures contained α-Τ and δ-Τ in the molar ratios shown in Table 9.

Analysis of variance (ANOVA) and lack-of-fit tests were used to determine the overall model significance (R^2^, *p*-value), as well as the significance of the model (equation) coefficients, at a minimum level of 95%. Additionally, a second-order polynomial model was used to predict the response variable as a function of the investigated independent components, as shown in Equation (3):

(3) $$Y_{k}{=\beta }_{0}+\overset{2}{\underset{i=1}{\sum }}\beta_{i}X_{i}+\overset{2}{\underset{i=1}{\sum }}\beta_{ii}X_{i}^{2}+\overset{2}{\underset{i=1}{\sum }}\overset{3}{\underset{j=i+1}{\sum }}\beta_{ij}X_{i}X_{j}$$

where *Y_k_* is the predicted response variable; *X_i_* and *X_j_* are the independent variables; and *β*_0_, *β_i_*, *β_ii_*, and *β_ij_* are the intercept and regression coefficients of the linear, quadratic, and interaction terms of the model, respectively. The greatest peak area and the effect of a significant independent variable on response were both determined using response surface methodology (RSM). The model equation was visualized using 3D surface response graphs.

### 3.4. DPPH Radical Scavenging Activity

Tocopherol samples were analyzed for their capacity to scavenge the 2,2-diphenyl-1-picrylhydrazyl radical (DPPH) in accordance with Kalantzakis et al. [39], with some modifications. Briefly, a 25 μL volume of tocopherol sample (in pure form or mixture) was mixed with 975 μL of DPPH solution (100 μM in ethyl acetate) and absorbance was read at 515 nm immediately after mixing (*A*_515(i)_) and after exactly 30 min (*A*_515(f)_), using a Shimadzu UV-1700 UV/vis spectrophotometer (Kyoto, Japan). The capacity to scavenge the DPPH radical was expressed as shown in Equation (4):

(4) $$\mathrm{Inhibition}\;\left(\%\right)=\left(\frac{A_{515(i)}-A_{515(f)}}{A_{515(i)}}\right)\;\times \;100$$

A calibration curve was prepared using Trolox (50–1500 μM) and the results were expressed as μM Trolox equivalent antioxidant capacity (TEAC).

After determining the optimum antioxidant mixture of tocopherols, experiments were repeated with the optimum combination to validate its efficiency. Moreover, in order to examine whether the observed effect was due to addition, antagonism or synergism, the individual tocopherols were also tested for their DPPH radical scavenging activity at the concentrations specified in the optimum mixture. The scavenging activity of the mixture was compared with the theoretical sum of the activities of the individual tocopherols.

### 3.5. Determination of Tocopherol Content of Oils

The method used for determination of tocopherol contents was a modification of the method reported by Lalas et al. [40]. The analysis was carried out on a Shimadzu CBM-20A (Shimadzu Europa GmbH, Duisburg, Germany) high-performance liquid chromatograph (HPLC) equipped with a SIL-20AC autosampler and a CTO-20AC column oven. Detection was carried out using a Shimadzu RF-10AXL fluorescence detector set to 294 nm (excitation) and 329 nm (emission). The column used was a Waters *μ*-Porasil (125 Å, 10 μm, 3.9 mm × 300 mm; Waters Corp., Milford, MA, USA). The mobile phase consisted of *n*-hexane/2-propanol/absolute ethanol (97.5:2.0:0.5, *v/v/v*) at a flow rate of 1 mL/min. The preparation of the sample was as follows: 0.25 g of oil was accurately weighed into a 5-mL volumetric flask, *n*-hexane was added and the mixture was shaken vigorously. A 20 μL sample was injected into the HPLC. Tocopherol content (TC) was determined as mg of each tocopherol per Kg of oil using the following equation:

(5) $$\mathrm{TC}\;(\mathrm{mg}\;T/\mathrm{Kg}\;\mathrm{Oil})=\frac{C_{T}\;\times \;V\;\times \;1000}{w}$$

where *C*_T_ is the concentration of each tocopherol (in mg/L), *V* is the volume of the extraction medium (in L) and *w* is the weight of the oil sample (in g).

### 3.6. Oil Fatty Acid Composition

The method for preparing the fatty acid methyl esters (FAMEs) from oils was according to Commission Regulation (EC) No 796/2002 (Annex XB) [41]. Analysis of methyl esters with GC-FID was carried out according to a modification of the method described by Lalas et al. [42]. An Agilent Technologies (Santa Clara, CA, USA) Gas Chromatograph model 7890A, equipped with an Omegawax capillary column (30 m × 320 μm × 0.25 μm) (Supelco, Bellefonte, PA, USA), was used. Helium was the carrier gas at a flow rate of 1.4 mL/min. The column temperature program was: initially isotherm for 5 min at 70 °C, ramped to 160 °C at a rate of 20 °C/min, then increased to 200 °C at a rate of 4 °C/min and increased up to 240 °C at a rate of 5 °C/min. The injector and flame ionization detector (FID) temperatures were maintained at 240 and 250 °C, respectively. The flow rate for hydrogen was 50 mL/min, for air 450 mL/min, and the makeup flow of helium 50 mL/min. Samples of 1.0 μL were injected in split mode (1:100). The individual peaks were identified by comparison with reference standards from FAME Mix C8–C24 (Sigma-Aldrich, St. Louis, MO, USA). The percentage composition of the samples was computed from the GC peak areas using the normalization method (without correction factors). The component percentages were calculated as mean values from triplicate GC-FID analysis.

### 3.7. Oil Oxidation Process

The oil oxidation process was carried out using a Rancimat 743 (Metrohm LTD, Herisau, Switzerland). More specifically, 10 g of each oil (as described in Table 10) was weighed in the Rancimat’s reaction vessels. Oxidation of the oils was carried out at 90 °C with 15 L/h airflow. After determination of the tocopherol content of the oils (as described in Section 3.5), the optimum mixture of tocopherols was prepared in situ by the addition of appropriate amounts of tocopherols. The oils were vortexed for 1 min and placed in the Rancimat for further oxidation. Blank samples were also prepared, without the addition of any tocopherol. Moreover, oils fortified with 200 ppm BHT were also examined as a positive control. A kinetic study was undertaken for the samples by measuring them every three hours (i.e., at 0, 3, 6, 9, 12, and 24 h). According to a previous report, 24 h of storage at 65 °C is equivalent to one month of storage at room temperature [43]. Therefore, storage for 24 h at an even more elevated temperature was expected to cause increased oxidation to oil samples.

### 3.8. Oil Quality Tests

#### 3.8.1. Peroxide Value (PV) Assay

The IDF standard method, 74A:1991 [44], was employed to determine the peroxide values of all oil samples with some modifications. Briefly, 0.05 g of oil sample was dissolved in a 2-mL Eppendorf tube with 2 mL dichloromethane/ethanol (3:2, *v/v*) on a vortex mixer for 2–4 s. Oil sample extract (20 μL) was mixed with 1960 μL of solvent (dichloromethane/ethanol). Ammonium thiocyanate solution (10 μL, 4 M in water) was added, and the sample was mixed on a vortex mixer for 2–4 s. Then, 10 μL of ammonium iron(II) sulfate solution (25.5 mM in 10 M HCl) was added, and the sample was mixed on a vortex mixer for 2–4 s. After 5 min incubation at room temperature, the absorbance of the sample was measured at 500 nm using a UV spectrophotometer against a blank solution (i.e., reaction mixture without lipid).

PV was determined using a hydrogen peroxide (H_2_O_2_) calibration curve that was constructed via repeating the above procedure at six different concentrations (50–500 μmoL/L in DCM/EtOH). PV obtained was expressed as mmoL H_2_O_2_ per Kg of oil, using the following equation:

(6) $$\mathrm{PV}\;\left(\mathrm{mmoL}\;H_{2}O_{2}/\mathrm{Kg}\;\mathrm{Oil}\right)=\frac{C_{H_{2}O_{2}}\;\times \;V}{w}$$

where $C_{H_{2}O_{2}}$ is the concentration of H_2_O_2_ (in μmoL/L), *V* is the volume of the extraction medium (in L) and *w* is the weight of the reaction oil sample (in g).

#### 3.8.2. Thiobarbituric Acid Reactive Substances (TBARS) Assay

The assay for TBARS determination was carried out according to Qiu et al. [45]. In a tube, 0.1 g of oil sample was added to 5 mL of TBA solution (prepared by mixing 15 g of trichloroacetic acid, 0.375 g of TBA, and 1.76 mL of 12 M HCl into a 100-mL volumetric flask, making up a final volume of 100 mL with deionized water). The mixture was shaken vigorously and incubated at 95 °C for 20 min. After incubation, samples were placed in an ice bath 5 min. Then, 200 μL of chloroform was added, and the mixture was vortexed and then centrifuged at 4500 rpm for 10 min. The absorbance of the supernatant was measured at 532 nm using a UV spectrophotometer. A blank solution was prepared by replacing the sample with deionized water. TBA value was determined as mmoL of malondialdehyde equivalents (mmoL MDAE) per Kg of oil, using a malondialdehyde calibration curve (15–300 µmoL/L in deionized water) according to the following equation:

(7) $${\mathrm{TBA}}_{\mathrm{value}}\;(\mathrm{mmoL}\;\mathrm{MDAE}/\mathrm{Kg}\;\mathrm{Oil})=\frac{C_{\mathrm{MDA}}\;\times \;V}{w}$$

where *C*_MDA_ is the concentration of malondialdehyde (in μmoL/L), *V* is the volume of the extraction medium (in L) and *w* is the weight of the oil sample (in g).

#### 3.8.3. *p*-Anisidine Value (*p*-AV) Assay

Anisidine value was determined using the ES ISO 6885:2012 method [46]. In a 10-mL volumetric flask, isooctane was added to 0.5 g of oil sample up to a final volume of 10 mL. From the diluted oil sample solution, 1 mL was transferred in a tube, and 0.2 mL of glacial acetic acid was added and shaken vigorously. After 10 min of incubation in the dark, absorbance was measured (*A*_0_) at 350 nm. In addition, 1 mL of the diluted oil sample solution was taken and added to 0.2 mL of *p*-anisidine analytical reagent (0.5% in glacial acetic acid) and shaken vigorously. After 10 min in the dark, the absorbance (*A*_1_) of the solution was measured at 350 nm. Then, 1 mL of isooctane was taken and added to 0.2 mL of *p*-anisidine analytical reagent, shaken vigorously and, after 10 min kept in the dark, absorbance was measured (*A*_2_) at 350 nm. *p*-AV was calculated using the expression:

(8) $$p\text{-AV}=\frac{100\;Q\;V}{m}\;0.24\left[{(A}_{1}-A_{2}-A_{0})\right]=12\left(\frac{A_{1}-A_{2}-A_{0}}{m}\right)$$

where *Q* is the sample content of the measured solution, in grams per milliliter (*Q* = 0.05 g/mL); *V* is the volume in which the test sample is dissolved, in milliliters (*V* = 10 mL); *m* is the mass of the test portion, in grams; *A*_0_ is the absorbance of the unreacted test solution; *A*_1_ is the absorbance of the reacted solution; *A*_2_ is the absorbance of the blank; 0.24 is the correction factor for the dilution of the test solution with 0.2 mL of reagent or glacial acetic acid (+20%).

#### 3.8.4. Conjugated Dienes and Trienes Determination

The method for measuring values of conjugated dienes and trienes was according to Pegg et al. [47]. In a 5-mL volumetric flask, 0.01 g of oil sample was added and cyclohexane was added. Absorbance was measured at 232 nm and 270 nm for conjugated dienes and trienes, respectively.

The conjugated diene (CD) and triene (CT) values were calculated using the following equations:

(9) $$C_{\mathrm{CD}}\;(\mathrm{mmoL}/\mathrm{mL})=\;\frac{A_{232}}{\varepsilon \;\times \;l}\;\;\;{\mathrm{CD}}_{\mathrm{value}}\;(\mathrm{mmoL}/\mathrm{Kg}\;\mathrm{Oil})=\frac{C_{\mathrm{CD}}{\;\times \;(5\;\times \;10}^{3})}{w}$$

(10) $$C_{\mathrm{CT}}\;(\mathrm{mmoL}/\mathrm{mL})=\frac{A_{270}}{\varepsilon \;\times \;l}\;\;\;{\mathrm{CT}}_{\mathrm{value}}\;(\mathrm{mmoL}/\mathrm{Kg}\;\mathrm{Oil})=\frac{C_{\mathrm{CT}}{\;\times \;(5\;\times \;10}^{3})}{w}$$

where *C*_CD_ and *C*_CT_ are the CD and CT concentrations, respectively, in M (molar concentration); *A*_232_ and *A*_270_ are the absorbances of the lipid solution at 232 nm and 270 nm, respectively; *ε* is the molar absorptivity of linoleic acid hydroperoxide (2.525 × 10^4^ M^−1^ cm^−1^); *l* is the path length of the cuvette in cm (1 cm); 5 × 10^3^ is a factor that encompasses the volume of solvent (5 mL) used to dissolve the oil sample; and *w* is the weight of the oil sample in g, so that the contents of CDs and CTs can be expressed in mmol per Kg of oil.

#### 3.8.5. Totox Value (TV) Assay

Totox value was calculated based on the method in Galanakis et al. [5]. Totox value (TV) is a measure of total oxidation, including primary and secondary oxidation products. It is a combination of PV and *p*-AV:

TV = 2 × PV + *p*-AV (11)

### 3.9. Statistical Analysis

Analyses were conducted in triplicate and the results are presented as means of triplicate determinations. The statistical significance of differences between mean values was assessed by a one-way analysis of variance (ANOVA) test; *p* < 0.05 was considered statistically significant. The experimental design for the response surface methodology and all associated statistics was accomplished with JMP™ 16.

## 4. Conclusions

To summarize, SO and OPO samples were exposed to accelerated oxidation conditions via Rancimat incubation. The addition of δ-tocopherol to SO and OPO samples can stabilize both oils and restrain their values of PV, TBARS, *p*-AV, CD and CT, and Totox. Specifically, Totox values of oil samples were significantly correlated with Rancimat incubation time, which could be utilized to predict Totox value in silico. Primary and secondary oxidation products were noticeably decreased, which is highly preferable in order to increase the shelf life of oils. The synergistic effect of α-T and δ-T homologs in oil samples was more obvious in the 7:1 mixture than in the 4:1 mixture. SO samples, which were more vulnerable to oxidation, benefited most from the synergism. Tocopherol content was also affected by synergism, as tocopherols became more resistant to the oxidation process. As for fatty acid composition, no statistically significant differences (*p* < 0.05) were measured in SFA, MUFA, and PUFA concentrations during oxidation. However, it should be underlined that tocopherol synergism remarkably reduced the ratio between omega-6 and omega-3 fatty acids, even more than the synthetic antioxidant BHT. Moreover, this effect was achieved by adding a minimal amount of δ-tocopherol (~24 ppm) to OPO to increase the amount of δ-tocopherol to the desired concentration. Therefore, a lower amount of antioxidants was added compared to 200 ppm BHT. Overall, it can be concluded that the use of tocopherol mixtures is a promising alternative option that can be further exploited by food industries to prolong the shelf life of vulnerable vegetable oils.

## Figures and Tables

**Table 1:** Measured and predicted TEAC values of α-T/δ-Τ mixtures, determined for individual design points.

Design Point	Independent Variables		Response (μM TEAC)	
	*X*_1_	*X*_2_	Measured	Predicted
1	1 (-1)	1 (-1)	348.3	350.3
2	1 (-1)	7 (1)	242.2	245.7
3	7 (1)	1 (-1)	497.8	503.3
4	7 (1)	7 (1)	365.3	372.3
5	1 (-1)	4 (0)	275.7	270.2
6	7 (1)	4 (0)	422.4	409.9
7	4 (0)	1 (-1)	466.7	459.2
8	4 (0)	7 (1)	351.9	341.4
9	4 (0)	4 (0)	359.7	372.4
10	4 (0)	4 (0)	367.1	372.4

**Table 2:** DPPH assay for individual tocopherols.

Tocopherols	*X*_1_	*X*_2_	Response (μM TEAC)
α-T (mg/L)	7	-	463.2 ± 5.0
δ-T (mg/L)	-	1	1.6 ± 0.3
*Theoretical sum*			*464.8*

**Table 3:** Tocopherol contents (mg/Kg oil ± SD) in sunflower oil and olive pomace oil samples. Inside rows with a specific superscript letter (e.g., a–c for α-T values and A–D for δ-T values) indicate a significant (*p* < 0.05) difference for each Rancimat incubation time.

***Sunflower oil***								
**Rancimat incubation time (h)**	**Control**		**7:1 α-T/δ-T**		**4:1 α-T/δ-T**		**200 ppm BHT**	
	**α-T**	**δ-T**	**α-T**	**δ-T**	**α-T**	**δ-T**	**α-T**	**δ-T**
0	459.06 ± 16.53 ^a^	7.98 ± 0.39 ^C^	466.47 ± 26.59 ^a^	68.61 ± 2.74 ^B^	462.55 ± 27.75 ^a^	116.94 ± 4.09 ^A^	462.26 ± 20.80 ^a^	8.09 ± 0.49 ^C^
3	406.71 ± 11.79 ^a^	7.85 ± 0.39 ^C^	428.08 ± 11.99 ^a^	63.71 ± 3.82 ^B^	359.22 ± 15.81 ^b^	105.45 ± 6.43 ^A^	429.17 ± 24.89 ^a^	7.87 ± 0.35 ^C^
6	370.47 ± 24.08 ^a^	7.23 ± 0.45 ^C^	381.19 ± 16.01 ^a^	60.19 ± 3.73 ^B^	308.18 ± 13.87 ^b^	104.09 ± 2.08 ^A^	370.00 ± 19.98 ^a^	7.76 ± 0.51 ^C^
9	294.42 ± 12.66 ^b^	6.91 ± 0.38 ^C^	340.87 ± 13.98 ^a^	58.56 ± 2.69 ^B^	273.35 ± 11.48 ^b^	102.21 ± 3.07 ^A^	349.77 ± 13.64 ^a^	7.64 ± 0.22 ^C^
12	223.14 ± 16.29 ^c^	6.82 ± 0.30 ^C^	262.90 ± 13.93 ^b^	56.52 ± 4.24 ^B^	242.52 ± 11.16 ^b,c^	100.19 ± 0.33 ^A^	326.46 ± 21.87 ^a^	7.50 ± 0.36 ^C^
24	199.00 ± 8.16 ^c^	5.96 ± 0.16 ^D^	252.72 ± 14.40 ^b^	45.62 ± 1.23 ^B^	195.68 ± 8.02 ^c^	91.68 ± 0.55 ^A^	314.97 ± 15.43 ^a^	7.26 ± 0.15 ^C^
***Olive pomace oil***								
**Rancimat incubation time (h)**	**Control**		**7:1 α-T/δ-T**		**4:1 α-T/δ-T**		**200 ppm BHT**	
	**α-T**	**δ-T**	**α-T**	**δ-T**	**α-T**	**δ-T**	**α-T**	**δ-T**
0	192.27 ± 5.58 ^a^	4.03 ± 0.11 ^C^	191.85 ± 11.13 ^a^	28.03 ± 0.81 ^B^	190.80 ± 9.35 ^a^	47.82 ± 2.92 ^A^	196.74 ± 6.49 ^a^	4.02 ± 0.25 ^C^
3	170.92 ± 10.94 ^a^	3.96 ± 0.13 ^C^	177.88 ± 11.56 ^a^	22.90 ± 1.08 ^B^	168.40 ± 6.74 ^a^	43.37 ± 2.34 ^A^	173.21 ± 8.66 ^a^	3.95 ± 0.12 ^C^
6	166.34 ± 9.15 ^a^	3.66 ± 0.09 ^C^	147.11 ± 10.59 ^b^	18.11 ± 0.62 ^B^	121.94 ± 8.05 ^c^	40.53 ± 2.15 ^A^	147.81 ± 10.49 ^b^	3.77 ± 0.17 ^C^
9	140.93 ± 4.09 ^a^	3.36 ± 0.19 ^C^	139.94 ± 7.56 ^a^	15.71 ± 0.42 ^B^	105.45 ± 3.27 ^b^	35.60 ± 1.32 ^A^	145.57 ± 3.35 ^a^	3.66 ± 0.23 ^C^
12	127.14 ± 6.87 ^a^	3.02 ± 0.06 ^C^	135.74 ± 4.62 ^a^	12.65 ± 0.70 ^B^	97.37 ± 6.91 ^b^	32.52 ± 0.19 ^A^	138.65 ± 8.87 ^a^	3.42 ± 0.26 ^C^
24	114.27 ± 3.20 ^b^	2.87 ± 0.19 ^D^	132.48 ± 5.03 ^a^	9.50 ± 0.19 ^B^	95.17 ± 2.38 ^c^	27.07 ± 0.07 ^A^	128.26 ± 4.87 ^a^	3.21 ± 0.12 ^C^

**Table 4:** Changes in the percentages of fatty acids in samples of sunflower oil and olive pomace oil during Rancimat incubation time (h). Inside columns with a specific superscript letter (e.g., a–d) indicate a significant (*p* < 0.05) difference for each Rancimat incubation time.

***Sunflower oil***								
**Oil samples**	**t (h)**	**∑ SFA ^1^**	**∑ MUFA ^2^**	**∑ PUFA ^3^**	**PUFA:SFA ratio**	**MUFA:PUFA ratio**	**ω-6:** **ω-3 ratio**	**COX ^4^**
Control	0	7.64 ± 0.41 ^a^	41.17 ± 1.66 ^a^	51.20 ± 3.31 ^a^	6.70 ± 0.08 ^a^	0.80 ± 0.02 ^a,b^	88.24 ± 2.56 ^a^	5.75 ± 0.36 ^a^
7:1 α-T/δ-T		7.63 ± 0.37 ^a^	40.57 ± 1.38 ^a^	51.80 ± 2.79 ^a^	6.78 ± 0.04 ^a^	0.78 ± 0.02 ^a,b^	89.32 ± 1.16 ^a^	5.80 ± 0.30 ^a^
4:1 α-T/δ-T		7.58 ±0.24 ^a^	40.23 ± 1.85 ^a^	52.20 ± 3.58 ^a^	6.88 ± 0.25 ^a^	0.77 ± 0.02 ^b^	89.98 ± 4.05 ^a^	5.84 ± 0.39 ^a^
200 ppm BHT		7.51 ± 0.30 ^a^	41.91 ± 0.26 ^a^	50.59 ± 2.84 ^a^	6.73 ± 0.11 ^a^	0.83 ± 0.04 ^a^	88.84 ± 0.80 ^a^	5.69 ± 0.30 ^a^
Control	3	7.22 ± 0.18 ^a^	36.41 ± 1.17 ^a^	56.37 ± 3.03 ^a^	7.81 ± 0.23 ^c^	0.65 ± 0.01 ^a^	122.89 ± 2.46 ^a,b^	6.22 ± 0.33 ^a^
7:1 α-T/δ-T		7.16 ± 0.43 ^a^	35.75 ± 0.71 ^a^	57.09 ± 1.39 ^a^	7.98 ± 0.29 ^c^	0.63 ± 0.01 ^a^	122.20 ± 4.29 ^a,b^	6.29 ± 0.15 ^a^
4:1 α-T/δ-T		6.39 ± 0.43 ^b^	35.39 ± 1.03 ^a^	58.22 ± 3.30 ^a^	9.12 ± 0.09 ^a^	0.61 ± 0.02 ^a^	117.83 ± 4.01 ^b^	6.41 ± 0.35 ^a^
200 ppm BHT		6.76 ± 0.47 ^a,b^	35.85 ± 0.27 ^a^	57.39 ± 3.56 ^a^	8.49 ± 0.06 ^b^	0.63 ± 0.03 ^a^	126.57 ± 0.89 ^a^	6.32 ± 0.37 ^a^
Control	6	7.55 ± 0.29 ^a^	35.31 ± 0.09 ^a^	57.14 ± 1.62 ^a^	7.57 ± 0.07 ^c^	0.62 ± 0.02 ^a^	124.19 ± 5.11 ^b^	6.29 ± 0.17 ^a^
7:1 α-T/δ-T		7.19 ± 0.44 ^a,b^	35.99 ± 1.98 ^a^	56.82 ± 1.50 ^a^	7.91 ± 0.27 ^b^	0.63 ± 0.02 ^a^	123.38 ± 5.08 ^b^	6.26 ± 0.18 ^a^
4:1 α-T/δ-T		6.94 ± 0.41 ^a,b^	36.26 ± 1.20 ^a^	56.80 ± 2.32 ^a^	8.19 ± 0.15 ^b^	0.64 ± 0.01 ^a^	123.96 ± 2.61 ^b^	6.26 ± 0.25 ^a^
200 ppm BHT		6.61 ± 0.38 ^b^	35.12 ± 0.11 ^a^	58.27 ± 3.79 ^a^	8.82 ± 0.07 ^a^	0.60 ± 0.04 ^a^	134.20 ± 0.54 ^a^	6.40 ± 0.39 ^a^
Control	9	7.38 ± 0.28 ^a^	35.32 ± 0.74 ^a^	57.30 ± 2.06 ^a^	7.76 ± 0.02 ^c^	0.62 ± 0.01 ^a,b^	135.41 ± 0.68 ^a^	6.30 ± 0.22 ^a^
7:1 α-T/δ-T		7.18 ± 0.48 ^a^	36.14 ± 0.90 ^a^	56.68 ± 1.27 ^a^	7.91 ± 0.35 ^b,c^	0.64 ± 0.00 ^a^	125.22 ± 5.78 ^b^	6.25 ± 0.14 ^a^
4:1 α-T/δ-T		7.05 ± 0.25 ^a,b^	35.14 ± 2.39 ^a^	57.81 ± 3.52 ^a^	8.20 ± 0.21 ^b^	0.61 ± 0.00 ^b^	127.45 ± 0.51 ^b^	6.36 ± 0.39 ^a^
200 ppm BHT		6.50 ± 0.15 ^b^	36.38 ± 0.15 ^a^	57.12 ± 2.18 ^a^	8.78 ± 0.13 ^a^	0.64 ± 0.02 ^a^	140.33 ± 2.53 ^a^	6.29 ± 0.23 ^a^
Control	12	7.16 ± 0.41 ^a^	35.15 ± 1.41 ^a^	57.69 ± 1.34 ^a^	8.07 ± 0.28 ^b^	0.61 ± 0.01 ^a,b^	143.41 ± 4.89 ^b^	6.34 ± 0.15 ^a^
7:1 α-T/δ-T		7.14 ± 0.29 ^a^	34.25 ± 2.19 ^a^	58.61 ± 2.64 ^a^	8.21 ± 0.04 ^a,b^	0.58 ± 0.01 ^b^	138.54 ± 0.42 ^b,c^	6.43 ± 0.30 ^a^
4:1 α-T/δ-T		6.98 ± 0.25 ^a^	35.85 ± 2.08 ^a^	57.17 ± 1.50 ^a^	8.19 ± 0.08 ^a,b^	0.63 ± 0.02 ^a^	134.72 ± 3.92 ^c^	6.29 ± 0.18 ^a^
200 ppm BHT		7.15 ± 0.48 ^a^	35.97 ± 0.17 ^a^	59.88 ± 3.30 ^a^	8.38 ± 0.10 ^a^	0.60 ± 0.03 ^a,b^	156.74 ± 3.15 ^a^	6.57 ± 0.34 ^a^
Control	24	7.26 ± 0.49 ^a^	32.24 ± 0.77 ^b^	60.51 ± 3.98 ^a^	8.34 ± 0.01 ^a^	0.53 ± 0.02 ^c^	157.94 ± 7.27 ^b^	6.60 ± 0.42 ^a^
7:1 α-T/δ-T		7.12 ± 0.48 ^a^	34.34 ± 1.58 ^a,b^	58.53 ± 1.41 ^a,b^	8.24 ± 0.36 ^a^	0.59 ± 0.01 ^b^	145.62 ± 2.77 ^c^	6.42 ± 0.16 ^a^
4:1 α-T/δ-T		7.05 ± 0.16 ^a^	35.89 ± 1.94 ^a^	57.06 ± 1.21 ^a,b^	8.09 ± 0.01 ^a^	0.63 ± 0.02 ^a^	135.07 ± 5.28 ^d^	6.28 ± 0.15 ^a^
200 ppm BHT		7.48 ± 0.37 ^a^	36.36 ± 0.23 ^a^	56.16 ± 1.29 ^b^	7.52 ± 0.20 ^b^	0.65 ± 0.01 ^a^	167.92 ± 0.17 ^a^	6.19 ± 0.14 ^a^
***Olive pomace oil***								
**Oil samples**	**t (h)**	**∑ SFA ^1^**	**∑ MUFA ^2^**	**∑ PUFA ^3^**	**PUFA:SFA ratio**	**MUFA:PUFA ratio**	**ω-6:** **ω-3 ratio**	**COX ^4^**
Control	0	12.47 ± 0.66 ^a^	73.75 ± 4.69 ^a^	13.78 ± 0.60 ^a^	1.10 ± 0.01 ^a^	5.35 ± 0.11 ^a^	18.75 ± 0.13 ^a,b^	2.23 ± 0.11 ^a^
7:1 α-T/δ-T		12.54 ± 0.81 ^a^	73.55 ± 4.81 ^a^	13.91 ± 0.99 ^a^	1.11 ± 0.01 ^a^	5.29 ± 0.03 ^a^	18.10 ± 0.38 ^b^	2.24 ± 0.15 ^a^
4:1 α-T/δ-T		12.47 ± 0.75 ^a^	73.65 ± 0.26 ^a^	13.88 ± 0.49 ^a^	1.11 ± 0.03 ^a^	5.31 ± 0.17 ^a^	18.91 ± 0.47 ^a^	2.24 ± 0.06 ^a^
200 ppm BHT		12.77 ± 0.32 ^a^	73.35 ± 4.16 ^a^	13.88 ± 1.03 ^a^	1.09 ± 0.05 ^a^	5.29 ± 0.09 ^a^	18.88 ± 0.36 ^a^	2.23 ± 0.15 ^a^
Control	3	12.69 ± 0.35 ^a^	67.72 ± 2.84 ^a^	19.66 ± 1.15 ^a^	1.55 ± 0.05 ^b^	3.45 ± 0.06 ^b^	19.24 ± 0.27 ^a^	2.81 ± 0.16 ^a^
7:1 α-T/δ-T		12.48 ± 0.41 ^a^	67.91 ± 3.53 ^a^	19.61 ± 1.39 ^a^	1.57 ± 0.06 ^b^	3.47 ± 0.07 ^b^	19.00 ± 0.46 ^a^	2.81 ± 0.18 ^a^
4:1 α-T/δ-T		12.20 ± 0.57 ^a^	68.19 ± 0.32 ^a^	19.61 ± 1.44 ^a^	1.61 ± 0.04 ^b^	3.49 ± 0.24 ^b^	19.03 ± 0.57 ^a^	2.81 ± 0.16 ^a^
200 ppm BHT		10.43 ± 0.51 ^b^	71.02 ± 5.08 ^a^	18.55 ± 0.69 ^a^	1.78 ± 0.02 ^a^	3.82 ± 0.13 ^a^	18.52 ± 0.00 ^a^	2.72 ± 0.13 ^a^
Control	6	12.59 ± 0.88 ^a^	66.95 ± 2.28 ^a^	20.46 ± 1.25 ^a^	1.63 ± 0.01 ^c^	3.28 ± 0.09 ^b^	19.99 ± 0.02 ^a^	2.89 ± 0.16 ^a^
7:1 α-T/δ-T		11.23 ± 0.40 ^b^	69.38 ± 4.89 ^a^	19.39 ± 1.20 ^a^	1.73 ± 0.05 ^b^	3.58 ± 0.03 ^a^	20.07 ± 0.06 ^a^	2.78 ± 0.18 ^a^
4:1 α-T/δ-T		12.69 ± 0.68 ^a^	67.59 ± 0.28 ^a^	19.72 ± 0.83 ^a^	1.55 ± 0.02 ^d^	3.43 ± 0.13 ^a,b^	19.64 ± 0.35 ^b^	2.81 ± 0.09 ^a^
200 ppm BHT		10.44 ± 0.30 ^b^	70.01 ± 4.42 ^a^	19.54 ± 0.78 ^a^	1.87 ± 0.02 ^a^	3.58 ± 0.08 ^a^	18.83 ± 0.02 ^c^	2.81 ± 0.13 ^a^
Control	9	12.06 ± 0.82 ^a,b^	68.52 ± 3.09 ^a^	19.42 ± 1.06 ^a^	1.61 ± 0.02 ^b^	3.53 ± 0.03 ^a^	22.67 ± 0.64 ^a^	2.77 ± 0.14 ^a^
7:1 α-T/δ-T		12.62 ± 0.82 ^a^	68.03 ± 4.48 ^a^	19.35 ± 0.80 ^a^	1.53 ± 0.04 ^c^	3.51 ± 0.09 ^a^	21.76 ± 0.39 ^b^	2.76 ± 0.13 ^a^
4:1 α-T/δ-T		12.8 ± 0.58 ^a^	67.66 ± 0.38 ^a^	19.54 ± 1.09 ^a^	1.53 ± 0.02 ^c^	3.47 ± 0.17 ^a^	22.00 ± 0.33 ^a,b^	2.78 ± 0.12 ^a^
200 ppm BHT		11.10 ± 0.76 ^b^	69.05 ± 4.80 ^a^	19.85 ± 1.41 ^a^	1.79 ± 0.01 ^a^	3.48 ± 0.01 ^a^	19.90 ± 0.04 ^c^	2.83 ± 0.20 ^a^
Control	12	11.76 ± 0.49 ^b,c^	69.48 ± 2.10 ^a^	18.76 ± 1.04 ^a^	1.59 ± 0.02 ^b^	3.71 ± 0.09 ^a^	32.48 ± 0.88 ^a^	2.68 ± 0.13 ^a^
7:1 α-T/δ-T		13.11 ± 0.94 ^a,b^	66.87 ± 4.12 ^a^	20.02 ± 1.33 ^a^	1.53 ± 0.01 ^b,c^	3.34 ± 0.02 ^c^	23.4 ± 1.01 ^b^	2.81 ± 0.18 ^a^
4:1 α-T/δ-T		13.27 ± 0.75 ^a^	66.93 ± 0.52 ^a^	19.80 ± 0.51 ^a^	1.49 ± 0.05 ^c^	3.38 ± 0.06 ^b,c^	22.34 ± 0.99 ^b,c^	2.80 ± 0.06 ^a^
200 ppm BHT		10.90 ± 0.72 ^c^	69.25 ± 2.03 ^a^	19.85 ± 0.42 ^a^	1.82 ± 0.08 ^a^	3.49 ± 0.03 ^b^	21.06 ± 0.17 ^c^	2.83 ± 0.07 ^a^
Control	24	10.44 ± 0.41 ^b^	70.16 ± 3.33 ^a^	19.40 ± 1.00 ^a^	1.86 ± 0.02 ^b^	3.62 ± 0.02 ^a^	74.53 ± 1.64 ^a^	2.72 ± 0.14 ^a^
7:1 α-T/δ-T		12.43 ± 0.63 ^a^	66.69 ± 4.42 ^a^	20.89 ± 1.09 ^a^	1.68 ± 0.00 ^d^	3.19 ± 0.05 ^b^	28.72 ± 0.17 ^c^	2.89 ± 0.16 ^a^
4:1 α-T/δ-T		11.97 ± 0.59 ^a^	66.87 ± 0.53 ^a^	21.16 ± 0.95 ^a^	1.77 ± 0.01 ^c^	3.16 ± 0.12 ^b^	24.30 ± 0.12 ^d^	2.93 ± 0.11 ^a^
200 ppm BHT		9.93 ± 0.55 ^b^	70.20 ± 2.68 ^a^	19.87 ± 0.87 ^a^	2.00 ± 0.02 ^a^	3.53 ± 0.02 ^a^	31.37 ± 0.72 ^b^	2.81 ± 0.12 ^a^

^1^ SFA, saturated fatty acids (%): SUM of C12:0, lauric acid; C14:0, myristic acid; C16:0, palmitic acid; C18:0, stearic acid; C20:0, arachidic acid; C22:0, behenic acid; C24:0, lignoceric acid. ^2^ MUFA, monounsaturated fatty acids (%): SUM of C16:1, palmitoleic acid; C18:1, oleic acid; C22:1, erucic acid. ^3^ PUFA, polyunsaturated fatty acids (%): SUM of C18:2, ω-6, linoleic acid; C18:3, ω-3, linolenic acid. ^4^ COX, Calculated Oxidizability Value.

**Table 5:** Peroxide values (mmoL H_2_O_2_/Kg Oil ± SD) of sunflower oil and olive pomace oil samples. Inside rows with a specific superscript letter (e.g., a–c) indicate a significant (*p* < 0.05) difference for each Rancimat incubation time.

***Sunflower oil***				
**Rancimat incubation time (h)**	**Control**	**7:1 α-T/δ-T**	**4:1 α-T/δ-T**	**200 ppm BHT**
0	7.56 ± 0.88 ^a^	6.36 ± 0.83 ^a^	7.13 ± 1.41 ^a^	6.01 ± 0.09 ^a^
3	17.09 ± 0.59 ^b^	14.01 ± 0.76 ^c^	19.78 ± 1.29 ^a^	13.77 ± 1.81 ^c^
6	28.82 ± 0.19 ^a,b^	26.67 ± 1.66 ^b,c^	30.44 ± 0.85 ^a^	25.06 ± 1.78 ^c^
9	42.92 ± 1.62 ^a^	42.48 ± 1.49 ^a^	43.46 ± 0.80 ^a^	33.68 ± 1.68 ^b^
12	62.01 ± 0.98 ^a^	55.70 ± 2.62 ^b^	58.23 ± 0.85 ^b^	45.33 ± 1.27 ^c^
24	82.43 ± 0.68 ^a^	77.39 ± 1.16 ^b^	81.79 ± 1.27 ^a^	63.14 ± 1.72 ^c^
***Olive pomace oil***				
**Rancimat incubation time (h)**	**Control**	**7:1 α-T/δ-T**	**4:1 α-T/δ-T**	**200 ppm BHT**
0	12.82 ± 1.32 ^a^	10.65 ± 1.05 ^b^	11.85 ± 1.15 ^a,b^	7.36 ± 0.87 ^c^
3	13.01 ± 0.79 ^a^	12.14 ± 0.90 ^a^	12.55 ± 0.64 ^a^	7.39 ± 0.17 ^b^
6	13.22 ± 0.27 ^a^	11.89 ± 0.80 ^a^	12.68 ± 0.60 ^a^	9.11 ± 1.75 ^b^
9	13.05 ± 0.79 ^a^	13.21 ± 0.57 ^a^	13.31 ± 0.82 ^a^	13.17 ± 1.24 ^a^
12	15.56 ± 0.19 ^a^	14.01 ± 0.39 ^b^	14.36 ± 0.91 ^b^	13.58 ± 0.66 ^b^
24	29.37 ± 1.09 ^a^	26.05 ± 1.01 ^b,c^	27.32 ± 1.05 ^b^	25.44 ± 0.23 ^c^

**Table 6:** TBARS values (mmoL MDAE/Kg Oil ± SD) of sunflower oil and olive pomace oil samples. Inside rows with a specific superscript letter (e.g., a–d) indicate a significant (*p* < 0.05) difference for each Rancimat incubation time.

***Sunflower oil***				
**Rancimat incubation time (h)**	**Control**	**7:1 α-T/δ-T**	**4:1 α-T/δ-T**	**200 ppm BHT**
0	0.48 ± 0.02 ^a^	0.44 ± 0.04 ^a^	0.24 ± 0.07 ^b^	0.30 ± 0.04 ^b^
3	0.79 ± 0.03 ^a^	0.73 ± 0.02 ^b^	0.36 ± 0.02 ^d^	0.53 ± 0.02 ^c^
6	0.91 ± 0.01 ^a^	0.88 ± 0.04 ^a^	0.47 ± 0.01 ^c^	0.73 ± 0.04 ^b^
9	1.17 ± 0.06 ^a^	0.78 ± 0.13 ^c^	0.52 ± 0.02 ^d^	0.96 ± 0.02 ^b^
12	1.50 ± 0.08 ^a^	0.81 ± 0.01 ^d^	1.41 ± 0.01 ^b^	1.19 ± 0.04 ^c^
24	2.15 ± 0.02 ^a^	1.62 ± 0.10 ^b^	1.68 ± 0.02 ^b^	1.47 ± 0.02 ^c^
***Olive pomace oil***				
**Rancimat incubation time (h)**	**Control**	**7:1 α-T/δ-T**	**4:1 α-T/δ-T**	**200 ppm BHT**
0	0.24 ± 0.01 ^b^	0.58 ± 0.02 ^a^	0.56 ± 0.08 ^a^	0.20 ± 0.04 ^b^
3	0.51 ± 0.01 ^b^	0.73 ± 0.02 ^a^	0.54 ± 0.07 ^b^	0.27 ± 0.04 ^c^
6	0.60 ± 0.02 ^b^	0.72 ± 0.04 ^a^	0.75 ± 0.03 ^a^	0.18 ± 0.01 ^c^
9	0.74 ± 0.09 ^b^	0.75 ± 0.04 ^a,b^	0.85 ± 0.05 ^a^	0.19 ± 0.01 ^c^
12	0.84 ± 0.01 ^b^	0.95 ± 0.09 ^a^	0.86 ± 0.03 ^a,b^	0.27 ± 0.02 ^c^
24	1.05 ± 0.02 ^a^	0.94 ± 0.10 ^b^	0.95 ± 0.04 ^a,b^	0.41 ± 0.01 ^c^

**Table 7:** *p*-Anisidine values (± SD) of sunflower oil and olive pomace oil samples. Inside rows with a specific superscript letter (e.g., a–c) indicate a significant (*p* < 0.05) difference for each Rancimat incubation time.

***Sunflower oil***				
**Rancimat incubation time (h)**	**Control**	**7:1 α-T/δ-T**	**4:1 α-T/δ-T**	**200 ppm BHT**
0	7.09 ± 0.11 ^b^	7.01 ± 0.06 ^b^	7.31 ± 0.09 ^a^	7.28 ± 0.07 ^a^
3	7.97 ± 0.09 ^a^	7.63 ± 0.02 ^b^	7.52 ± 0.10 ^b^	8.09 ± 0.10 ^a^
6	8.02 ± 0.11 ^b,c^	7.93 ± 0.09 ^c^	8.46 ± 0.10 ^a^	8.19 ± 0.14 ^b^
9	8.25 ± 0.11 ^b^	8.11 ± 0.20 ^b^	8.55 ± 0.08 ^a^	8.55 ± 0.19 ^a^
12	8.90 ± 0.09 ^a^	8.33 ± 0.07 ^b^	8.81 ± 0.17 ^a^	8.70 ± 0.08 ^a^
24	9.42 ± 0.19 ^a^	9.00 ± 0.21 ^b^	8.83 ± 0.17 ^b^	8.75 ± 0.09 ^b^
***Olive pomace oil***				
**Rancimat incubation time (h)**	**Control**	**7:1 α-T/δ-T**	**4:1 α-T/δ-T**	**200 ppm BHT**
0	5.75 ± 0.08 ^a,b^	5.49 ± 0.11 ^b,c^	5.82 ± 0.22 ^a^	5.26 ± 0.18 ^c^
3	6.06 ± 0.10 ^a^	6.07 ± 0.09 ^a^	5.92 ± 0.07 ^a^	5.50 ± 0.09 ^b^
6	6.12 ± 0.01 ^b^	7.02 ± 0.10 ^a^	5.91 ± 0.12 ^c^	6.03 ± 0.09 ^b,c^
9	6.57 ± 0.12 ^b^	7.36 ± 0.04 ^a^	6.62 ± 0.13 ^b^	6.60 ± 0.13 ^b^
12	7.07 ± 0.13 ^b^	7.66 ± 0.10 ^a^	6.82 ± 0.07 ^c^	6.71 ± 0.15 ^c^
24	7.35 ± 0.17 ^a,b^	7.62 ± 0.16 ^a^	6.97 ± 0.30 ^b,c^	6.85 ± 0.17 ^c^

**Table 8:** Conjugated dienes (CD) and trienes (CT) values (mmol/Kg oil ± SD) in sunflower oil and olive pomace oil samples. Inside rows with a specific superscript letter (e.g., a–d for CD values and A–D for CT values) indicate a significant (*p* < 0.05) difference for each Rancimat incubation time.

***Sunflower oil***								
**Rancimat incubation time (h)**	**Control**		**7:1 α-T/δ-T**		**4:1 α-T/δ-T**		**200 ppm BHT**	
	**CD**	**CT**	**CD**	**CT**	**CD**	**CT**	**CD**	**CT**
0	17.33 ± 0.13 ^a^	12.88 ± 0.16 ^A^	14.34 ± 0.58 ^b,c^	10.11 ± 0.41 ^C^	13.73 ± 0.27 ^c^	9.90 ± 0.23 ^C^	14.78 ± 0.15 ^b^	12.00 ± 0.14 ^B^
3	19.78 ± 0.27 ^a^	10.03 ± 0.23 ^C^	20.11 ± 0.07 ^a^	10.40 ± 0.03 ^B^	18.28 ± 0.20 ^b^	10.58 ± 0.11 ^B^	18.44 ± 0.23 ^b^	11.23 ± 0.17 ^A^
6	22.10 ± 0.15 ^c^	9.24 ± 0.09 ^D^	23.86 ± 0.21 ^a^	10.03 ± 0.11 ^B^	23.50 ± 0.18 ^b^	10.98 ± 0.08 ^A^	20.19 ± 0.04 ^d^	9.79 ± 0.03 ^C^
9	25.85 ± 0.07 ^b^	8.69 ± 0.02 ^D^	29.75 ± 0.06 ^a^	9.95 ± 0.04 ^B^	29.78 ± 0.07 ^a^	9.57 ± 0.06 ^C^	24.91 ± 0.26 ^c^	11.39 ± 0.18 ^A^
12	34.05 ± 0.26 ^b^	9.67 ± 0.17 ^B^	35.91 ± 0.07 ^a^	9.84 ± 0.08 ^B^	36.14 ± 0.23 ^a^	9.78 ± 0.05 ^B^	28.28 ± 0.14 ^c^	10.85 ± 0.13 ^A^
24	42.49 ± 0.65 ^a^	10.36 ± 0.21 ^C^	40.71 ± 0.05 ^b^	9.72 ± 0.07 ^D^	39.81 ± 0.03 ^c^	10.79 ± 0.10 ^B^	37.83 ± 0.07 ^d^	12.18 ± 0.09 ^A^
***Olive pomace oil***								
**Rancimat incubation time (h)**	**Control**		**7:1 α-T/δ-T**		**4:1 α-T/δ-T**		**200 ppm BHT**	
	**CD**	**CT**	**CD**	**CT**	**CD**	**CT**	**CD**	**CT**
0	17.45 ± 0.28 ^b^	4.34 ± 0.14 ^B^	17.80 ± 0.06 ^a^	4.55 ± 0.06 ^A^	17.44 ± 0.08 ^b^	4.13 ± 0.05 ^C^	16.43 ± 0.03 ^c^	4.01 ± 0.04 ^C^
3	17.48 ± 0.57 ^a^	4.37 ± 0.05 ^A^	17.26 ± 0.75 ^a,b^	4.46 ± 0.18 ^A^	16.44 ± 0.05 ^b^	3.91 ± 0.03 ^B^	16.94 ± 0.11 ^a,b^	4.06 ± 0.03 ^B^
6	17.60 ± 0.09 ^a^	4.58 ± 0.05 ^A^	17.37 ± 0.51 ^a^	4.46 ± 0.05 ^A^	17.48 ± 0.36 ^a^	4.15 ± 0.12 ^B^	16.67 ± 0.07 ^b^	4.00 ± 0.10 ^B^
9	17.97 ± 0.23 ^a^	4.46 ± 0.07 ^B^	17.57 ± 0.08 ^b^	4.63 ± 0.07 ^A^	17.11 ± 0.06 ^c^	4.10 ± 0.02 ^C^	16.94 ± 0.11 ^c^	4.03 ± 0.06 ^C^
12	17.87 ± 0.24 ^c^	4.58 ± 0.16 ^A,B^	18.42 ± 0.08 ^b^	4.73 ± 0.07 ^A^	18.93 ± 0.16 ^a^	4.52 ± 0.14 ^B^	15.69 ± 0.03 ^d^	4.03 ± 0.03 ^C^
24	18.06 ± 0.06 ^c^	4.86 ± 0.03 ^A^	19.79 ± 0.34 ^a^	4.61 ± 0.10 ^B^	18.70 ± 0.21 ^b^	4.15 ± 0.04 ^C^	17.12 ± 0.03 ^d^	4.16 ± 0.11 ^C^

**Table 9:** Experimental values and coded levels of the independent variables used in the 3 × 3 factorial design.

Independent Variables	Code Units	Coded Variable Level		
		−1	0	1
α-T/δ-Τ mixture				
α-T (mg/L)	*X*_1_	1	4	7
δ-Τ (mg/L)	*X*_2_	1	4	7

**Table 10:** Oil samples (blank and fortified) oxidized with the Rancimat.

Sunflower Oil (SO) Samples	Olive Pomace Oil (OPO) Samples
SO (blank)	OPO (blank)
SO with 7:1 α-T/δ-Τ mixture	OPO with 7:1 α-T/δ-Τ mixture
SO with 4:1 α-T/δ-T mixture	OPO with 4:1 α-T/δ-T mixture
SO spiked with 200 ppm BHT	OPO spiked with 200 ppm BHT

## Data Availability

Not applicable.
